# Corrosion-Fatigue Failure of Gas-Turbine Blades in an Oil and Gas Production Plant

**DOI:** 10.3390/ma13040900

**Published:** 2020-02-18

**Authors:** Mojtaba Rajabinezhad, Abbas Bahrami, Mohammad Mousavinia, Seyed Jalil Seyedi, Peyman Taheri

**Affiliations:** 1 Department of Materials Engineering, Isfahan University of Technology, Isfahan 84156-83111, Iran; mojtabaa.rajabi22@gmail.com (M.R.); a.n.bahrami@iut.ac.ir (A.B.); 2 Inspection and Corrosion Engineering Department, Pars Oil and Gas Company (POGC), Assalouyeh 1414713111, Iran; m.mousavinia1986@gmail.com; 3 National Iranian South Oilfields Company (NISOC), South Turbine Maintenance Centeral Shop, Omidieh 61735-1333, Iran; jalils198925s@gmail.com; 4 Department of Materials Science and Engineering, Delft University of Technology, Mekelweg 2, 2628 CD Delft, The Netherlands

**Keywords:** gas turbine, corrosion-fatigue, blades, failure

## Abstract

This paper investigates the root cause of a failure in gas-turbine blades, made of Nimonic-105 nickel-based superalloy. The failure was reported in two blades in the second stage of a turbine-compressor of a gas turbine in the hot section. Two failed blades were broken from the root and from the airfoil. The failure took place after 20 k h of service exposure in the temperature range 700–850 °C, with the rotating speed being in the range 15,000–16,000 rpm. The microstructures of the failed blades were studied using optical/electron microscopes. Energy dispersive X-ray spectroscopy (EDS) was employed for phase identification. Results showed that failure first initiated from the root. The dominant failure mechanism in the root was concluded to be corrosion-fatigue. The failure scenario was suggested based on the results obtained.

## 1. Introduction

Gas-turbine blades are known to be extremely critical and important components in power plants. They are expected to operate in harsh working conditions, such as high temperature, high pressure and complex dynamic loading conditions. Degradation and failure of gas-turbine blades can obviously have severe negative implications for the integrity and functionality of gas turbines and hence the output of a power plant. Failures of gas-turbine blades can be due to creep damage, high-temperature corrosion, high-temperature oxidation, fatigue, erosion, and foreign object damage [1,2,3,4,5,6,7,8,9,10,11,12,13,14,15]. Obviously, in most cases the failure is due to the interrelation of more than one failure mechanism [2]. As a case in point, creep and fatigue can simultaneously result in failure in gas-turbine blades [2]. Increasing the operating temperature in gas turbines has always been a target, given that any increase in service temperature is associated with an increase in the efficiency and output of a turbine.

Among different candidate materials for harsh high-temperature working conditions in turbines, nickel- and cobalt-based superalloys have long been known as the best options on the grounds that these alloys are capable of working at high temperature, while they keep their properties [16]. These alloys benefit from a unique and exceptional combination of high-temperature mechanical properties (i.e., superior creep resistance), excellent resistance to high-temperature degradation/oxidation, and superior microstructural stability during high-temperature service [17]. Nickel-based superalloys are extensively used in critical components in aerospace and gas-turbine engines. In fact, about 50 wt.% of aero/gas turbine-engines are made of nickel-based superalloys [18]. Other applications of nickel-based superalloys include chemical/petrochemical plants, high-temperature reactors, and marine applications. Different grades of nickel-based superalloys are now currently used in different applications, amongst which Inconel 718 is one of the most widely used. Other widely used grades include FGH95, ME-16, RR1000, IN-100, Udimet 720LI, Nimonic 80A, Inconel 825, Nimonic C-263, Nimonic-75, and Nimonic-105 [19]. The latter alloy is extensively used in hot combustion chambers of gas turbines [20], with service temperatures being as high as 950 °C. Nimonic-105 is often cast in air. For more demanding and sensitive applications, vacuum melting is highly recommended.

This research investigates a failure in gas-turbine blades in the second stage of a turbine-compressor of a gas turbine (i.e., the gas producer or hot section) in an oil and gas production plant. The failed blades were made of Nimonic-105 nickel-based superalloy. The main focus in this investigation is on the microstructural and metallurgical aspects of the failure. Even though the consequences of failures in the third row might not be as severe as those in the first and second rows, they need to be evaluated and analyzed carefully. Failures of blades in a gas turbine are often catastrophic in the sense that this causes severe damages in the turbine, associated with a significant economic loss. Therefore, it is necessary to conduct a failure investigation to analyze the root cause of failure and eventually to mitigate similar failures in any oil- and gas-production and power plants. This certainly has positive implications for the system reliability and service life of a gas turbine. Lessons learnt from a failure study will certainly be useful in planning failure mitigation strategies in similar plants/set-ups [21,22,23,24,25,26,27,28,29,30]. It will be shown that the failure, reported in this study, is a corrosion-fatigue failure. Given that not so many corrosion-fatigue failures have been reported in the literature, this manuscript will certainly be useful, especially when it comes to analyzing/controlling the same form of damage in similar gas turbines.

## 2. Experimental

The microstructural analyses in this case were performed using scanning electron microscopy (SEM, Philips, Eindhoven, The Netherlands) and optical microscopy (OM, Nikon, Japan). Energy-dispersive X-ray spectroscopy (Philips, The Netherlands) was employed to study chemical compositions of different phases in the microstructure. Failed blades were first visually evaluated. Macro images were then taken by Stereo Microscope Nikon SMZ 800 (Nikon, Tokyo, Japan). For optical microscopy, samples were first ground with grinding papers. Samples were then polished with diamond paste (3 and 1 μm). Fractography was also performed on failed specimens to determine dominant failure mechanisms.

## 3. Case Background

This failure is related to a gas turbine where its input air volume can be as high as 5220 m^3^/h. Approximately 40% of this sucked-in air is used for combustion. The sucked-in air pressure is close to 100 psi. The combustion air to fuel ratio is 15. The fuel is a methane/ethane gas mixture which has been dried before entering into the gas turbine. The failure in this case was in blades in the second stage in which 69 blades are assembled on a disk. Based on the available specification, blades are made of Nimonic-105. Nickel-based rotating blades are expected to operate under extreme working conditions, where erosion and corrosion phenomena come into play. Besides, blades in nickel-based superalloys in aggressive working conditions are expected to show great resistance against cavitational erosion. Blades in the second stage are expected to have service life over 100 k h up to 120 k h. In this case, blades failed after approximately 20 k h service, which makes this failure a typical premature incident. A major drawback of having a blade fail in a whole assembly is that all the blades have to be replaced with brand new blades or blades with similar service lifetime. This has to do with the fact that it is a routine practice to make sure all blades are in similar conditions in terms of service life. This makes the interpretation of non-destructive testing (NDT) results much easier. The working temperature in the hot section of the turbine is 700–850 °C. The rotating speed of the blades is in the range 15,000–16,000 rpm. The blades are made of Nimonic-105 nickel-based superalloy with the following chemical composition (Table 1).

## 4. Results and Discussion

### 4.1. Visual Observations

**Figure 1:**
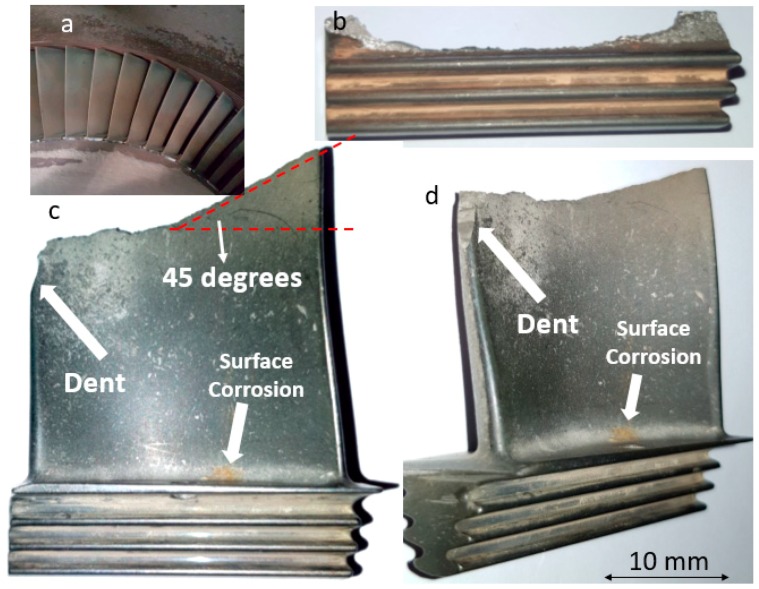
Visual observation of broken blade, (**a**) and overview of blades in the assembled condition, (**b**) failed blade from the root, and (**c**,**d**) broken blade from the airfoil.

The reported failure in this case was related to two turbine blades as shown in Figure 1, with one failing from the root and the other from the airfoil. These two blades were located in the vicinity of each other. There are a few observations worth mentioning. First of all, there was no severe surface corrosion on either side of the blades, except for a brownish spot shown in Figure 1. This does not appear to be a severely corroded spot. Also, there was no excessive deformation (i.e., noticeable thinning or buckling) in the profile of the failed airfoil. One can also see that there was no surface irregularity observed in the failed blades. This includes surface cracking or pitting. In one edge of the failed airfoil, one can see a dent, which seems to be due to the collision of a high-energy flying object. The last important visual observation to mention is that the fracture in the airfoil has two areas: the flat surface and the part with 45-degree inclination. This is clearly shown in Figure 1c.

### 4.2. Microstructure of Failed Blades

**Figure 2:**
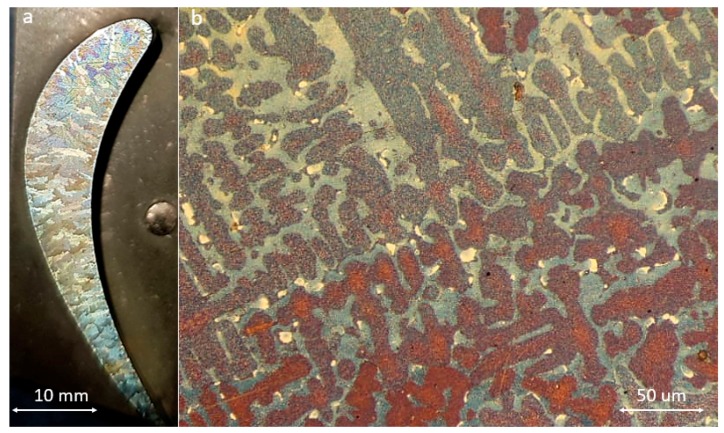
(**a**) Macro and (**b**) optical microscope images of failed blades.

Figure 2 depicts the typical microstructure in the failed blades. The microstructure is a typical dendritic cast structure where dendrites have grown from two sides towards the centerline of the blade, as expected. Fine white particles in-between dendrites are carbide particles.

### 4.3. Fractography of Failed Blades

**Figure 3:**
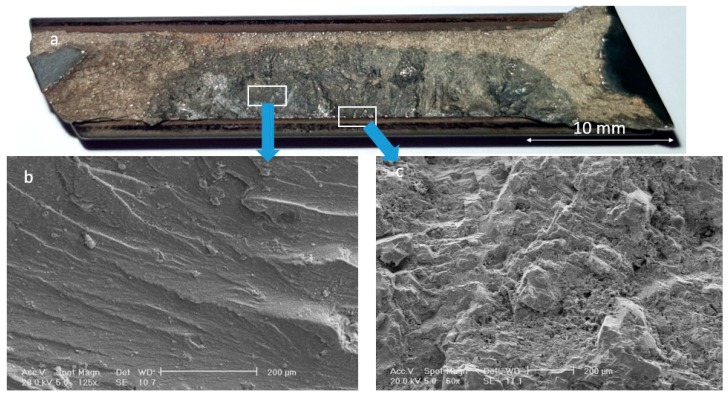
(**a**) Stereomicroscope and (**b**,**c**) scanning electron microscope (SEM) micrographs of the fracture surface of the blade, failed from the root.

**Figure 4:**
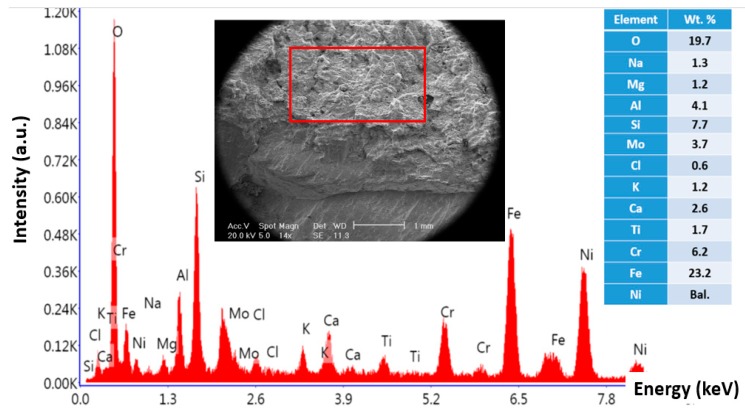
Energy dispersive X-ray spectroscopy (EDS) analysis of corrosion products at the edge of broken blade.

Figure 3 shows stereomicroscope and SEM micrographs of the fracture surface from the blade that failed from the root. As shown in Figure 3a, the fracture surface has two areas: the dominant grayish area and the rest of the surface. This is a typical fracture surface when fatigue is the governing damage mechanism. Figure 3b,c show SEM micrographs of two areas, highlighted in Figure 3a. The former image (Figure 3b) shows typical beach marks, indicating that fatigue is an active mechanism. Further down to the edge, there is a clear change in the fracture surface (Figure 3c). The surface at the edge of the failed blade is covered with some deposits, which were analyzed by EDS. Results of EDS analysis of the microstructure at the edge (presented in Figure 4) showed that surface deposits are rich in Cl, K, Na, and Ca. This, together with the fact that there is a high concentration of oxygen in the analysis, indicates that these deposits are in fact corrosion products. It is highly likely that the aforementioned elements come from the fuel/gas.

**Figure 5:**
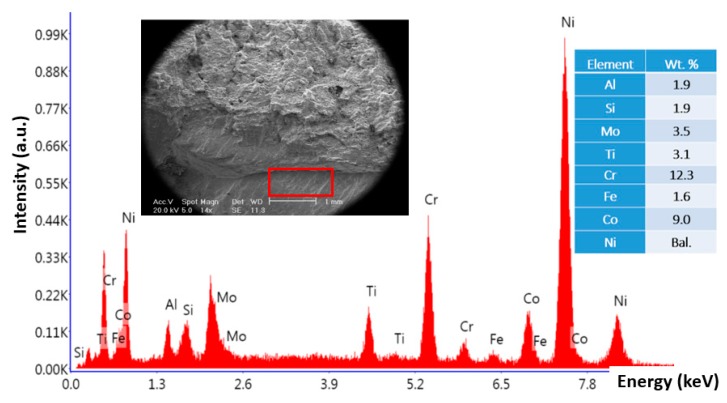
EDS analysis of the area with beach marks in the failed blade, broken from the root.

Results of EDS analysis of the microstructure further away from the edge is presented in Figure 5. There is hardly any oxygen, Na, Ca, K, and Cl in the analysis, inferring that the aforementioned corroded area is limited to few hundred micrometers from the edge.

From the results obtained, it can be concluded that the failure in the blade, broken from the root, was controlled by the simultaneous activation of corrosion and fatigue. Corrosion-fatigue is a common degradation mechanism in moving components exposed to high-temperature harsh working conditions [21]. Corrosion fatigue essentially takes place under the synergistic activation of corrosion and dynamic loading. This degradation mechanism is one of the most common reasons for the failure of turbine blades [22]. Corrosion fatigue is undoubtedly one of the most complex failure mechanisms. Both mechanical and chemical aspects of failure in this case are equally important. Any mitigation measure to avoid this failure necessitates a multi-disciplinary approach. Corrosion-fatigue failure of the blade root in this case has possibly originated from corrosion pits/products deposited at the root/airfoil interface. The results showed that corrosion products/deposits in this case mostly contained CaO, Na_2_O, KCl, and NaCl. The sensitivity of turbine blades to corrosion-fatigue initiation largely depends on the integrity of the passive film on the surface [22]. The breakdown of this passive film together with the deposition of oxide/corrosion deposits create an ideal condition for corrosion-fatigue crack initiation. The film breakdown and deposition of corrosion products causes anodic/cathodic sites, leading to an accelerated localized anodic dissolution reaction and, hence, localized corrosion [23]. The formation of corrosion-induced surface irregularities creates stress riser centers on the surface [24]. In case these surface irregularities exceed a critical size, a fatigue crack is likely to initiate. The crack then propagates under dynamic loading condition, finally leading the failure of the turbine blade.

**Figure 6:**
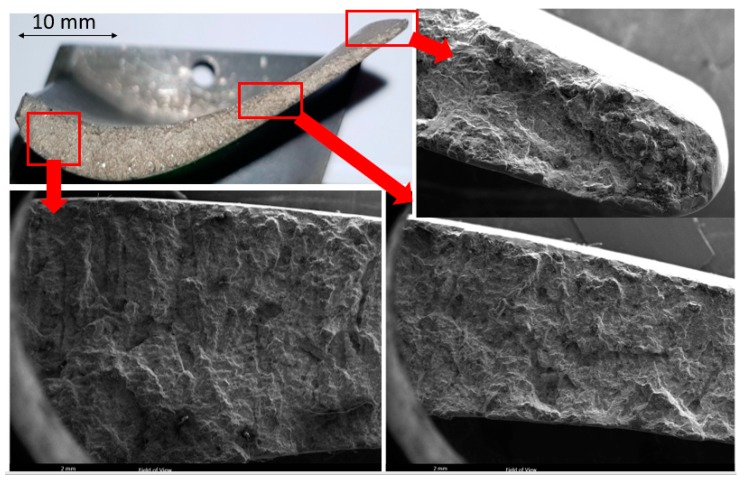
Images of fracture surface of the blade, broken from the airfoil.

**Figure 7:**
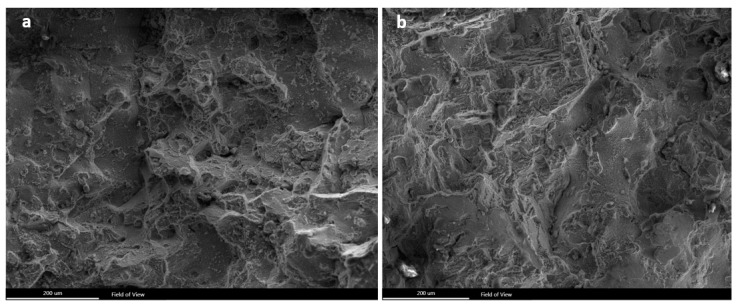
(**a**,**b**) SEM micrographs of fracture surface of the blade, broken from the airfoil.

Figure 6 shows stereomicroscope/SEM micrographs of the fracture surface of the sample, failed from the airfoil (see Figure 1c,d). SEM micrographs, shown from different areas, indicate that there is hardly any difference between fracture modes in different areas. More importantly, there is absolutely no indication that fatigue has been an active mechanism in this blade, i.e., there is no beach mark on the fracture surface. Figure 7 shows SEM micrographs of the fracture surface with higher magnification, again confirming that there is no fatigue involved in this case. Fracture in this sample has elements of ductile and brittle fractures. There are some areas with colonies of dimples and some areas with cleavage fracture. The fact that there is no fatigue in this blade is indicative that this blade has fractured due to a sudden overloading and/or collision. The logical failure scenario is that the blade that failed from the root has first undergone a corrosion-fatigue failure. The broken piece has then hit the adjacent blade, causing the fracture of the blade from the airfoil. The dent observed at the edge of the airfoil (highlighted in Figure 1c,d) has possibly formed as a result of a collision between the flying broken piece and the airfoil.

## 5. Concluding Remarks

This paper investigates a failure in gas-turbine blades made of Nimonic-105 nickel-based superalloy. Two broken blades were investigated, with one having failed from the root and the other from the airfoil. Blades are from the second row of the gas producer/hot section of a gas turbine with working temperature being in the range 700–850 °C and rotating speed in the range 15,000–16,000 rpm. The failure was reported after 20 k hours of service, which is much shorter than the anticipated service life. The following conclusions can be drawn, based on the data obtained:

− Nimonic-105 superalloy in this case had a typical dendritic cast structure, with dendrites growing from the edges towards the center.
− There were no surface irregularities, excessive deformation, buckling, or surface cracking observed on the surface of the blades, except for a dent on the edge of one of the broken blades.
− Results showed that corrosion fatigue is the governing failure mechanism in the blade that failed from the root. In this case, there are corrosion deposits at the edge of the blade, rich in Cl, Ca, O, Na, and K. Fatigue cracks have initiated from corroded spots at the edge of the blade.
− Fractography of the blade that failed from the airfoil showed that, contrary to the former case, there is hardly any fatigue involved in the failure of this blade.
− It was concluded that the blade that failed from the root first underwent a corrosion-fatigue failure, finally ending up in the breakage of the airfoil from the root. The broken piece then hit the adjacent blade in its trajectory, causing the fracture of the adjacent blade from the airfoil. The dent observed at the edge of the airfoil possibly formed as a result of the collision between the flying broken piece and the airfoil.
− Given that both corrosion and fatigue mechanisms were involved in this failure, it is important to minimize mechanical vibrations and to reduce contaminants in the system. The latter necessitates further investigation on the origins of the Cl, Na, and Ca-containing residues and how they can be controlled/eliminated from the system.

## Figures and Tables

**Table 1:** Chemical composition of Nimonic-105 nickel-based superalloy.

Element	C	Si	Cu	Fe	>Mn	Cr	Ti	Al	Co	Mo	Zr	Ni
**wt. %**	0.1	0.5	0.1	0.5	0.6	15.0	1.0	4.5	19.0	5.0	0.1	Bal.
